# Ultrasonographic Monitoring in 38 Dogs with Clinically Suspected Acute Pancreatitis

**DOI:** 10.3390/vetsci7040180

**Published:** 2020-11-16

**Authors:** Federico Puccini Leoni, Tina Pelligra, Simonetta Citi, Veronica Marchetti, Eleonora Gori, Caterina Puccinelli

**Affiliations:** Veterinary Teaching Hospital “Mario Modenato”, Department of Veterinary Sciences, University of Pisa, Via Livornese Lato Monte, 56121 Pisa, Italy; puccini.federico@yahoo.it (F.P.L.); pelligratina@gmail.com (T.P.); veronica.marchetti@unipi.it (V.M.); eleonora.gori@vet.unipi.it (E.G.); caterina.puccinelli@phd.unipi.it (C.P.)

**Keywords:** acute pancreatitis, abdominal ultrasound, specific pancreatic lipase, dog, comparison

## Abstract

Abdominal ultrasound examinations (AUEs) are commonly used in the diagnostic evaluation of canine acute pancreatitis (AP). The purpose of this retrospective study was to evaluate and monitor the ultrasonographic changes observed in dogs with clinically suspected AP on consecutive AUEs. The study population was constituted by 38 client-owned dogs hospitalized for no less than 48 h from January 2016 to December 2019. Dogs included in this study were suspected of AP based on the clinical examination and abnormal rapid specific canine pancreatic lipase test performed at admission. Dogs were submitted to two AUEs, the first on the first day of hospitalization, and the second between 40–52 h after the first one. Twelve dogs had both AUEs suggestive of AP. Fourteen dogs received an ultrasonographic diagnosis of AP exclusively on the second AUE. Twelve dogs remained negative on both the first and the second AUE. In 26 out of 38 patients the second AUE was suggestive of AP. If a patient is suspected of AP, it is advisable to carry out ultrasonographic monitoring at least within the first 52 h after admission, since ultrasonographic signs of AP may only become observable later after hospitalization.

## 1. Introduction

Pancreatic disorders are not rare events in dogs, and pancreatitis is the most common disease of the exocrine pancreas. Acute pancreatitis (AP) consists in a generally sterile inflammation with acute onset, which does not permanently destroy the architecture of the organ [1,2]. In fact, it can be reversible, unless the initial triggers of disease persist and chronic or recurrent inflammation ensues [1,2]. Clinical signs of AP in dogs vary greatly according to the severity and include anorexia, vomiting, weakness, abdominal pain, and diarrhea [2,3]. The results of a complete blood count, serum biochemistry profile and urine analysis in dogs are not specific of AP and thus cannot be considered diagnostic [4,5].

Canine pancreatic lipase immunoreactivity (cPLI) assay is currently considered the most sensitive and specific serum test for the diagnosis of AP in dogs because it exclusively measures lipase of pancreatic origin [5,6,7,8,9,10,11]. The Spec cPL^a^ assay is an enzyme-linked and commercially available immunoassay developed to quantitatively measure serum concentrations of specific canine pancreatic lipase (cPL) [5,7,11]. The normal reference interval for Spec cPL is 0 to 200 μg/L. Concentrations of ≥400 μg/L are highly suggestive of AP, while concentrations between 201 and 399 μg/L are non-diagnostic [5].

The estimated sensitivity and specificity of the Spec cPL assay for the diagnosis of AP has been reported to range respectively between 87–94% and 66–77% using a cutoff value of 200 μg/L, and between 72–78% and 81–88% using a cutoff value of 400 μg/L [11]. Three necropsy studies have shown a specificity between 90–100% for serum cPL using the recommended cut-off of 400 μg/L [1,9,10]. 

Although a new lipase activity assay (DGGR) has been validated for use in dogs showing a high sensitivity (93%) for AP and providing a similar performance to cPLI, a SNAP cPL test has been developed [5,12]. This is a rapid, in-clinic, semi-quantitative, less expensive, and visually readable assay for the evaluation of canine pancreatic lipase concentration in serum [13]. This SNAP cPL test consists in a version of the Spec cPL assay using SNAP ELISA technology and provides two possible results: normal or abnormal. If the SNAP cPL test is abnormal, the effective cPL concentration can be located in the gray zone, (between 200 and 400 μg/L) or in the zone corresponding to the diagnosis of AP (≥400 μg/L) [13]. For the diagnosis of canine AP, the sensitivity and specificity of the SNAP cPL test is between 92–94% and 71–78%, respectively [11]. The in-clinic SNAP cPL test provides accurate and reliable results, is easy to interpret, agrees with the Spec cPL assay, and enables the practitioner to determine with a high degree of confidence whether the level of pancreas-specific lipase in serum is normal or abnormal, and initiate immediate treatment [13]. Abdominal ultrasound examination (AUE) is the most commonly used diagnostic imaging technique and is the method of choice for the diagnosis of AP; it is also used to rule out other diseases that may cause similar clinical signs [5]. 

In 1998, Hess reported that the sensitivity of AUE for the diagnosis of AP in dogs was 68% [3]. However, a recent study showed that the sensitivity and specificity of AUE vary widely according to the number of ultrasonographic alterations taken into consideration: pancreatic enlargement, pancreatic abnormal echogenicity, or altered mesenteric echogenicity [14]. If only one of these three alterations was observed, the sensitivity and specificity of AUE for the detection of AP were 89% and 43% respectively. If two of the alterations were observed, the sensitivity and specificity were 78% and 69% respectively. On the other hand, when all three alterations were observed at the same time, the sensitivity and specificity were 43% and 92% respectively [14]. Therefore, the AUE does not enable a definitive diagnosis of AP and a normal pancreas on AUE does not exclude AP in dogs. 

Furthermore, when there is a clinical suspicion of AP and the initial AUE is within normal limits, it should be carried out again after 2–4 days, since the severity of ultrasonographic changes may increase over time [15]. A recent study subjected dogs with AP to two consecutive CT exams, in order to assess the presence of new alterations or a deterioration of the pancreatitis [16]. However, there are no studies in veterinary medicine that evaluate the ultrasonographic signs suggestive of AP to serial AUE [14]. The aim of the present study was to evaluate the ultrasonographic changes observed in dogs with clinically suspected AP and an abnormal SNAP cPL test result during AUE monitoring. 

## 2. Materials and Methods 

We carried out a retrospective analysis evaluating the database of all the canine patients admitted to the Veterinary Teaching Hospital “Mario Modenato” of the University of Pisa, from January 2016 to December 2019, in order to identify a population of dogs that met the following criteria:

- Clinical presentation consistent with AP. Dogs had to present at least two of the following clinical signs: vomiting, diarrhea, weakness, anorexia, abdominal pain [4],
- Hospitalization for 48 h or more,
- Abnormal SNAP cPL test at admission,
- At least two AUEs performed during the first three days of hospitalization, precisely: the first one on the first day of hospitalization (T0) and the second one on the third day of hospitalization (T1).

The SNAP cPL test consists of a reference spot that corresponds to the upper limit of the reference interval, and a sampling spot that is visually compared with the reference spot [13]. The results of this rapid test can be considered normal if the color of the sampling spot is less intense than the reference spot, or abnormal if the sampling spot appears as equal to, or more intense than, the reference spot. All AUEs were performed by the same two experienced radiologists (S.C. and T.P.). DICOM images and video files were stored in our hospital database and retrospectively reviewed by a third operator (F.P.L.). The AUE images were obtained using a Toshiba Aplio 400 (Canon Medical Systems Europe B.V., Zoetermeer, The Netherlands) with a 7.5 MHz microconvex probe and a 12 MHz linear probe. 

The following parameters were evaluated and considered as ultrasonographic highly suggestive alterations (HSAs) of AP: pancreatic diffuse or focal enlargement, pancreatic hypoechogenicity, pancreatic inhomogeneous echostructure, peripancreatic hyperechoic mesentery, and peripancreatic free fluid [17,18,19]. Wall thickening of the stomach and/or duodenum, signs of paralytic ileus with hypomotility and liquid pattern in the content, corrugated appearance of the duodenum and/or adjacent transverse colon, extrahepatic cholestasis with dilation of the common bile duct, dilation of the pancreatic duct, presence of pseudocysts or abscess, and venous thrombosis were also evaluated and considered as ultrasonographic possible suggestive alterations (PSAs) of AP [17,18,19,20]. The AUE was considered suggestive for AP only if at least two HSAs were observed.

Based on the onset time of HSAs, we classified the dogs into four groups: group A (dogs presenting at least two HSAs on both T0 and T1), group B (at least two HSAs only on T0), group C (at least two HSAs only on T1), group D (dogs not presenting at least two HSAs on both T0 and T1). 

Data were analyzed using a commercial statistical software (IBM SPSS Statistics, version 25, IBM Corporation, New York, NY, USA). The prevalence between T0 and T1 of HSAs and PSAs in group A and group C were compared using McNemar test. A *p*-value < 0.05 was considered statistically significant. 

## 3. Results

A total of 38 canine patients met the criteria required for inclusion. The study population consisted of 10 females (26.3%), of which four were sterilized, and 28 males (73.7%), of which three were castrated. Seven dogs were cross-breeds, while other canine patients belonged to the following breeds: Dachshund (*n* = 3), Beagle (*n* = 3), Golden Retriever (*n* = 2), English Setter (*n* = 2), Bracco (*n* = 2), Boxer (*n* = 2), Shih Tzu (*n* = 2), Siberian Husky (*n* = 1), Labrador (*n* = 1), Australian Shepherd (*n* = 1), Bolognese (*n* = 1), Pitt Bull (*n* = 1), Greyhound (*n* = 1), Toy Poodle (*n* = 1), Cocker Spaniel (*n* = 1), Jack Russel Terrier (*n* = 1), Bouvier des Flandres (*n* = 1), Border Collie (*n* = 1), Italian Spinone (*n* = 1), Cavalier King Charles (*n* = 1), Standard Poodle (*n* = 1), and Maltese (*n* = 1). The median age of the patients was nine years, with a variable range of between 5 months and 15 years.

The most common clinical signs were weakness (76%), anorexia (66%), vomiting (53%), diarrhea (34%), and abdominal pain (16%). All T0 were performed within six hours of admission, while all T1 were performed between 40–52 h after T0. On T0, 14 dogs (12 belonging to group A and two belonging to group C) showed 51 signs (38 HSAs + 13 PSAs) suggestive of AP, while on T1 26 dogs (all patients belonging to group A and group C) showed 105 signs (80 HSAs + 24 PSAs) suggestive of AP. As for group A (constituted by 12 dogs), three dogs presented additional HSAs on 

T1 compared to T0; eight dogs presented one or more PSAs on T0, and nine dogs presented one or more PSAs on T1. Table 1 summarizes the results listed above.

**Figure 1:**
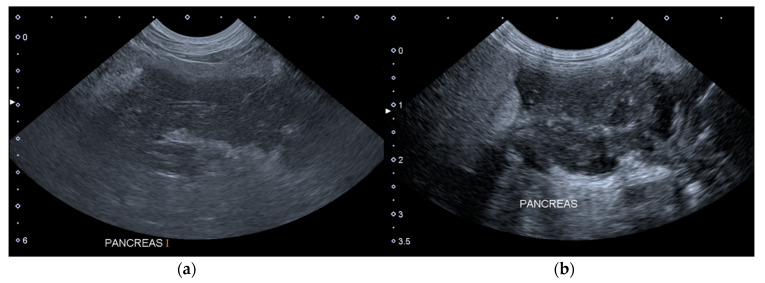
Longitudinal ultrasound images of the right lobe of the pancreas of a dog included in the group A. (**a**) T0: the pancreas is enlarged, hypoechoic, slightly inhomogeneous and with peripancreatic hyperechoic mesenteric fat. (**b**) T1: the pancreas is more inhomogeneous and with peripancreatic free fluid. T0: abdominal ultrasound examination performed on the first day of hospitalization; T1: abdominal ultrasound examination performed on the third day of hospitalization.

The Figure 1 shows two ultrasound images of the pancreas of a dog included in the Group A, which presented one additional HAS on T1.

**Figure 2:**
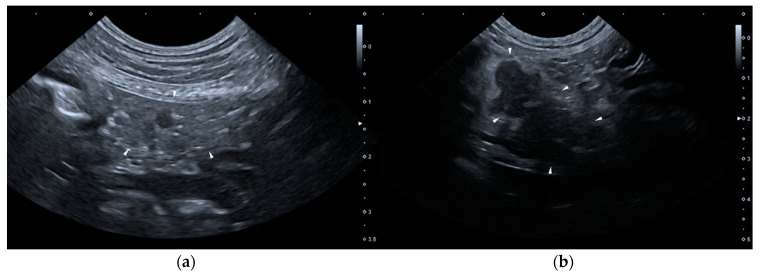
Ultrasound images of the right lobe of the pancreas of a dog included in the group C. (**a**) T0: transverse sonogram of the pancreas, which is normal in size, shape, echogenicity, and echostructure; (**b**) T1: longitudinal sonogram of the pancreas, which is slightly enlarged and inhomogeneous, hypoechoic and with peripancreatic hyperechoic mesenteric fat. T0: abdominal ultrasound examination performed on the first day of hospitalization; T1: abdominal ultrasound examination performed on the third day of hospitalization.

None of the dogs met the required criteria for group B. As for group C (constituted by 14 dogs), on T0 none of the dogs presented HSAs, but two dogs presented two or more PSAs: one of them presented wall thickening and corrugated appearance of duodenum and paralytic ileus, the other one showed wall thickening of the stomach, paralytic ileus and dilation of the common bile duct. Six dogs belonging to group C presented one or more PSAs on T1. Figure 2 shows two ultrasound images of the pancreas of a dog included in the Group C, of T0 and T1, respectively. 

As for group D (constituted by 12 dogs), none of the dogs presented signs (HSAs or PSAs) of AP on both T0 and T1. 

AUE was suggestive of AP in 26 out of 38 patients (68%); of these 26 dogs, 14 showed HSAs exclusively on T1. Among these 26 patients, pancreatic hypoechogenicity, and peripancreatic hyperechoic mesentery were the most common HSAs, and each one was observed in 22 out of 38 dogs on T1, while the least represented HSA was pancreatic enlargement. Dilation of the pancreatic duct and pancreatic pseudocysts or abscesses were observed just on T1, while no signs of venous thrombosis were detected. We observed a lower number of PSAs compared to HSAs in group A on both T0 and also T1, and in group C just on T1. All HSAs and PSAs observed on T0 and T1 were reported in Table 2.

No significant differences in T0 and T1 prevalence of HSAs and PSAs in group A and C were found (all *p* > 0.05).

## 4. Discussion

We evaluated a population of dogs with clinically suspected AP to determine whether repeated AUE is necessary to evaluate the progression of AP. The results highlighted an increase or a new appearance of ultrasonographic signs of AP between 40–52 h after T0. Although AUE is a routine examination for the imaging diagnosis of AP in dogs, in humans, contrast enhanced computed tomography is considered the gold standard for the diagnosis and staging of AP [5,21,22]. The veterinary literature reports that a CT performed at the time of admission is likely to be sufficient in the diagnosis and evaluation of dogs with AP, however it is highly improbable that repeated CT examinations add additional information in the absence of worsening clinical signs [21].

Based on the hypothesis formulated in a veterinary study according to which the findings observable on the AUE may vary based on the time elapsing since the onset of AP, we analyzed four groups [15]. The dogs belonging to group A had an AUE positive for AP on both T0 and T1. In these dogs, the visible ultrasonographic macroscopic damage induced by the AP probably occurred early, allowing an immediate visualization of the ultrasonographic alterations. It cannot be excluded that dogs belonging to this group were presented to the veterinary hospital some days after from the onset of AP, and therefore the time necessary for the microscopic damage to become ultrasonographically evident had already passed. In group A, pancreatic hypoechogenicity and peripancreatic free fluid were evident in the same number of dogs on both T0 and T1, while we observed an increase in other HSAs between T0 and T1.

The dogs belonging to group C showed a clinical presentation consistent with AP and an abnormal result at the SNAP cPL test; however, they had an AUE positive for AP only on T1. We hypothesize that these dogs had not yet developed such extensive damage on T0, which instead manifested itself on T1. This was assumed from the pathophysiology of AP which shows initial acinar cell damage that could subsequently affect the entire organ and also the peritoneal cavity [4]. We hypothesize that at hospital admission, there was microscopic cellular damage in these dogs, which resulted in a release of cPL in the blood circulation which in turn led to the abnormal SNAP cPL test result. In these 14 dogs, the AUE became positive between the first and the second checks as hypothesized by Hecht and Henry, and similarly to humans, where only in 20% of cases the pancreas demonstrates signs of AP on AUE before 48–72 h after symptom onset [15,23]. 

In human medicine, imaging has a limited role in the early phase of AP because pancreatic necrosis may be missed or misdiagnosed on contrast enhanced computed tomography in the first 72 h, since it can be confused with a diminished perfusion due to edema [22,24,25].

In group C, on T0 we observed a paralytic ileus associated with the wall thickening of the stomach or duodenum and the corrugated appearance of the duodenum in two dogs, one of which also showed the dilation of common bile duct. Based on their medical records, these two dogs suffered from chronic enteropathy, thus we hypothesized that these ultrasonographic alterations may be related to their underlying pathology. On T1, dogs belonging to group C showed the same type of HSA detected in group A. The dogs belonging to group D did not present ultrasonographic findings suggestive of AP either on T0 or T1. We assume that the pancreatic microscopic damage did not have enough time to hesitate in an ultrasonographic visible macroscopic alteration or that there was limited damage that only affected a small number of pancreatic acinar cells. 

The results of the SNAP cPL test are considered abnormal if the sampling spot appears equal to or more intense than the reference spot; however, in this case, the effective cPL concentration can be located in the gray zone or in the zone corresponding to the diagnosis of AP [13]. Therefore, in the absence of a quantitative examination test, the abnormal result could indicate a cPL concentration of between 200 and 400 μg/L, which is difficult to interpret as AP. This could correspond to probable pancreatic damage not markedly significant to hesitate in an ultrasonographic alteration. The human literature reports that in early or mild cases of AP, abnormal ultrasound findings can be seen in between 33% and 90% of patients with AP [26]. 

The limitations of our study include the lack of histopathological examinations to confirm the diagnosis of AP and the low number of patients. Histopathology is considered the gold standard for the diagnosis of AP in dogs; however, it is difficult to perform due to its invasive nature and pancreatic biopsy during AP may further injure the pancreas [16]. Many studies have used a variety of data to diagnose AP including clinical signs, clinicopathologic alterations, and cPL concentrations, and AUE has been used as a surrogate gold standard for the diagnosis of AP in dogs [11,14,27]. The limited dog population probably contributed to determine the absence of significant differences in T0 and T1 prevalence of HSAs and PSAs in group A and C.

Another limitation is that after performing the SNAP cPL test and reading abnormal results, the Spec cPL reference quantitative assay was not executed, because of the retrospective nature of our evaluation. However, there is a 90–100% agreement between the SNAP cPL test and the Spec cPL assay [13,28,29]. Finally, since the dogs were not subjected to the Spec cPL assay or to the histological examination, it is also possible that these dogs were not really affected by AP. For these reasons we do not assessed the sensitivity and specificity values of abdominal ultrasound for AP, since in other studies, either the histological examination [3] or the quantitative Spec cPL test [14] were performed in order to provide those values. Despite the significant agreement with the Spec cPL, we do not believe that the SNAP test gives such strong confidence. No signs of venous thrombosis were detected, however portal vein thrombosis is a severe sequela of AP, and it has been reported that CT is significantly more effective in detecting the presence of portal vein thrombosis than the AUE [16,21]. Portal vein thrombi are not commonly detected by AUE probably because the portal vein is located in a deep area of the cranial abdomen and because a lot of patients that undergo AUE are in severe pain or have other pathologic changes associated with AP that complicate the evaluation of the abdomen [21].

## 5. Conclusions

Although in the absence of statistical significance, in our study we observed in many patients a growth trend from T0 to T1 for the number of almost all the parameters considered (HSAs and PSAs), suggesting a worsening of the ultrasonographic appearance of AP between 40–52 h after T0. In conclusion, if a patient is suspected of AP, in addition to the evaluation of clinical signs and immunoserological tests, it is advisable to carry out ultrasonographic monitoring at least within the third day of hospitalization, since ultrasonographic alterations may become visible only after a few days.

## Figures and Tables

**Table 1:** The present table shows the total amount of HSAs and PSAs respectively observed in each group of dogs and in all the population of dogs on both T0 and T1

Groups	T0 Dogs (*n*)	T0 HSAs (*n*)	T0 PSAs (*n*)	T1 Dogs (*n*)	T1 HSAs (*n*)	T1 PSAs (*n*)
Group A (12 dogs)	12	38	8	12	44	15
Group C (14 dogs)	2	0	5	14	36	9
Group D (12 dogs)	0	0	0	0	0	0
Total (A + C + D)	14	38	13	26	80	24

**Table 2:** Ultrasonographic alterations suggestive of AP observed in group A, C, and D on T0 and on T1. AP: acute pancreatitis; T0: abdominal ultrasound examination performed on the first day of hospitalization; T1: abdominal ultrasound examination performed on the third day of hospitalization; NA: not applicable due to low sample size.

Ultrasonographic Alterations of AP	Group A (12 Dogs)		Group C (14 Dogs)		Group D (12 Dogs)	
	T0 *n* (%)	T1 *n* (%)	T0 *n* (%)	T1 *n* (%)	T0 *n* (%)	T1 *n* (%)
Pancreatic enlargement	5 (42%)	8 (76%)	0 (0%)	2 (14%)	0 (0%)	0 (0%)
Pancreatic hypoechogenicity	11 (92%)	11 (92%)	0 (0%)	11 (79%)	0 (0%)	0 (0%)
Pancreatic inhomogeneous echostructure	7 (58%)	9 (75%)	0 (0%)	4 (29%)	0 (0%)	0 (0%)
Peripancreatic hyperechoic mesentery	8 (67%)	9 (75%)	0 (0%)	13 (93%)	0 (0%)	0 (0%)
Peripancreatic free fluid	7 (58%)	7 (58%)	0 (0%)	6 (43%)	0 (0%)	0 (0%)
Wall thickening of the stomach or duodenum, corrugated appearance of duodenum	5 (42%)	7 (58%)	2 (14%)	3 (21%)	0 (0%)	0 (0%)
Paralytic ileus	3 (25%)	6 (50%)	2 (14%)	4 (29%)	0 (0%)	0 (0%)
Dilation of the common bile duct	0 (0%)	1 (8%)	1 (7%)	1 (7%)	0 (0%)	0 (0%)
Dilation of the pancreatic duct	0 (0%)	1 (8%)	0 (0%)	1 (7%)	0 (0%)	0 (0%)
Pancreatic pseudocysts or abscess	0 (0%)	0 (0%)	0 (0%)	0 (0%)	0 (0%)	0 (0%)
Venous thrombosis	0 (0%)	0 (0%)	0 (0%)	0 (0%)	0 (0%)	0 (0%)
